# A Study of Diurnal Cortisol Adaptations in Sleep-Deprived Firefighters During a 72-Hour Work Shift: A Case Series

**DOI:** 10.7759/cureus.37504

**Published:** 2023-04-12

**Authors:** Neil Sundberg, Richard M Millis

**Affiliations:** 1 Department of Pathophysiology, American University of Antigua, St. John's, ATG

**Keywords:** sleep deprivation, obesity, circadian rhythm, diurnal variation, salivary cortisol, fire fighters

## Abstract

Seventy percent of US firefighters are overweight or obese. The combination of sleep deprivation and exposure to traumatic events during 72-hour work shifts, commonly employed in emergency responders, is thought to put firefighters at high risk for a variety of stress-related diseases and suicide. Previous studies suggest that the cortisol awakening response (CAR) may be increased in sleep-deprived emergency responders. This case series was designed to investigate the variations in CAR and associations with measurements of salivary cortisol and testosterone, blood glucose and triglyceride, and blood pressure during a 72-hour work shift. Measurements were made at 08:00 and 20:00 in five participants (one normal weight normotensive, three obese hypertensive, and one morbidly obese normotensive male). Data were characterized by the regression statistic R^2^ computed from the relationship between diurnal measurement and concentration, with significance at R^2^≥0.4. The predominant AM CAR adaptation response consisted of no significant 72-h change (flat response) in salivary cortisol (R^2^<0.4), found in three of the five participants (60%). The normal-weight participant’s 72-h AM CAR adaptation was characterized as incremental (R^2^=0.91), and markedly different than that of the four obese firefighters who exhibited either a flat response (R^2^<0.4, 60%) or, in one subject, a decremental response (R^2^=0.40, 20%). The predominant 72-h PM cortisol adaptation was found to be decremental (R^2^=0.78-0.97) in three of the five participants (60%), including the normal weight subject (R^2^=0.78). Diurnal salivary cortisol and testosterone exhibited normal physiological circadian variations (P=0.01, AM>PM and P=0.1, AM>PM, respectively). Blood glucose and triglyceride also showed physiological circadian variations (P=0.02 AM<PM and P=0.002 AM<PM, respectively). Diurnal variation of systolic BP was found to be not significant (P=0.2). The flat AM CAR adaptation exhibited in three of four obese hypertensive firefighters may represent a blunted adaptation response, akin to the responses reported for survivors of suicide. These findings suggest that diurnal variations in salivary cortisol and testosterone, blood glucose, and triglyceride may be useful biochemical markers for identifying stress-related adaptations to 72-hour work shifts. Future studies should be designed to correlate diurnal variations in biomarkers with the risk of developing stress-related diseases and suicide in firefighters.

## Introduction

The hypothalamic-pituitary-adrenal (HPA) axis provides humans with a first-line emergency response system and the basis for physiological adaptations to stress. Exposures to environmental stressors that are particularly prolonged and/or intense often result in pathophysiological maladaptation [1,2]. Such dysfunctions involve HPA axis hyperactivity and overproduction of cortisol with blunted HPA axis responsiveness which appear to be important factors in the development of depression [3,4]. Depression, chronic stress, and continued HPA axis maladaptation, in turn, lead to various potentially lethal stress-related diseases such as overweight and obesity, metabolic syndrome, diabetes mellitus, hypertension, and heart failure [5,6]. That 70% of the US firefighter workforce is reported to be overweight or obese [7] suggests that comparing the HPA axis response characteristics of normal weight with those of overweight or obese subjects might help identify critical differences and factors which may inform the development of an effective intervention to decrease the prevalence of firefighter suicide, targeting the highest risk individuals. The elevated risk for the development of post-traumatic stress disorder, suicidal ideations, and other stress-related disorders by emergency responders (e.g., firefighters, emergency medical services, law enforcement officers, military personnel) produces significant morbidity and mortality [8]. Virtually all emergency responders work shifts on a 12, 24, 48, or 72-hour basis wherein their occupational stress is inextricably linked to a combination of sleep deprivation, arduous physical activity, and physical exhaustion. This combination of stressors increases cortisol and pro-inflammatory cytokines release, thereby explaining a positive correlation between inflammatory markers associated with cortisol, stress, and feelings of fatigue [9]. The diurnal variation in HPA axis activity associated with the cortisol awakening response (CAR), a predictable increase in plasma or salivary cortisol level within the first hour of awakening [10], is shown to be disrupted by shift work [11]. However, the effects of shift work on the CAR adaptations associated with frequent sleep disruption and exposure to traumatic events during a continuous 72-hour work shift have not been studied. This case series is designed to demonstrate the patterns in salivary cortisol adaptations and to evaluate their association with changes in salivary testosterone, plasma triglyceride glucose, and blood pressure, known mediators of stress, in a cohort of firefighters during the same 72-hour work shift.

## Case presentation

The Institutional Review Board of California State University Long Beach approved all research procedures. Informed consent was emailed to and completed by participants prior to data collection. Participants were recruited by a flyer from the population of firefighters in a Southern California battalion. Respondents were provided opportunities to ask questions via email or telephone contact. After questions were answered, an informed consent form was emailed, and a time was scheduled to review and discuss the consent form via telephone. We confirmed that the participants understood the procedures prior to consenting. Once the participants were comfortable with the consent form and study procedures, the participants signed the consent form, scanned it, and emailed it back to us. No compensation was provided to the participants.

This case series was designed to study the cortisol responses of firefighters from the same fire engine company during the same 72-h shift. Therefore, all procedures had to be performed within a Southern California engine company utilizing 72-h shifts. The small size of the case series is reflective of the unique logistical and financial challenges associated with original research in this field. We recruited five volunteers sharing the same 72-h shift because there were only five firefighters/firefighter paramedics assigned to the same shift in this duty station (three engine apparatus personnel and two ambulance personnel). Inclusion criteria consisted of full-time, permanent personnel who were able to understand, speak, read, and write the English language. Exclusion criteria prohibited participants from having a history, diagnoses, and/or prescription medication for a mental health disorder or medical conditions such as recent injuries or trauma that could potentially independently contribute to changes in salivary cortisol concentrations. Table 1 presents the demographic, physiological, and work shift-related characteristics of the participants.

**Table 1:** Characteristics of participants. Note: BMI fails to adequately reflect lean muscle mass which is why skin folds (via the well-validated 7-site Jackson Pollock method) and bioelectrical impedance analysis (BIA) were utilized. While BMI may be an appropriate measurement tool for an inactive/sedentary population these firefighters, although obese, engage in arduous physical activity on a daily basis with both physical training and firefighting training.

Subject Characteristics	Mean ± SD
Age (years)	29.6 ± 6.8
Height (in)	71.8 ± 1.79
Weight (lbs)	264.96 ± 64.84
Body mass index (BMI, kg/m^2^)	36.12 ± 9.04
Body index of adiposity (BIA, fat %)	29.64 ± 12.97
Skinfold adiposity (fat %)	26.72 ± 7.63
Waist-to-hip ratio (WHR)	0.9 ± 0.11
Resting heart rate	79 ± 8
Resting systolic blood pressure (mmHg)	129 ± 6
Resting diastolic blood pressure (mmHg)	81 ± 14
Years in fire service	6.9 ± 7.75
Hours worked prior to study shift	72 ± 79.6
Hours worked in last 30 days	331.2 ± 186.98

The participants were all non-smokers, and in accordance with their mandated fire department-wide injury illness prevention program, they were required to perform 1 hour of PT (physical training) per day while on duty. All participants were compliant with this standard and reported performing approximately 1 hour of PT/day outside of work on days off during personal time; although the nature of physical activity varied among subjects. All the subjects drank socially and denied a history of alcohol abuse or department disciplinary action for violation of drug or alcohol policy. All assigned to the same firefighting apparatus consumed identical meals at identical times. Subjects between apparatus (i.e. fire engine vs ambulance) had only the slightest difference in meal composition and timing (generally associated with additional time committed to the incident by ambulance personnel transporting patients from the scene of an event to the nearest hospital, then returning to the fire station).

Capillary blood was drawn for glucose, HbA1c, and lipid profiles (Cholestec LDX, Kernersville, NC). Saliva samples were assayed for cortisol and testosterone at the Salimetrics Saliva Lab (Carlsbad, CA) using the Salimetrics Salivary Testosterone Assay Kit (Cat. No. 1-2402), and Salimetrics Salivary Cortisol Assay Kit (Cat. No 1-3002), without modifications to the manufacturer’s protocol. We first ran a wet lab protocol for salivary cortisol and testosterone ELISAs in an unblinded manner to ensure appropriate results. Samples were then sent to a private lab (Salimetrics) for independent verification where lab personnel was blinded to all samples. Cortisol sensitivity was 0.007 g/dL, coefficient of variation 4.6-6.0%, and standard curve range 0.012-3.0 g/dL. Testosterone sensitivity was 1 pg/mL, coefficient of variation 4.60-9.85%, and standard curve range of 6.1-600 pg/mL. Participants were asked to rate their level of stress on a scale of 1-10. Table 2 summarizes the study group’s baseline blood and salivary measurements. The Likert scale used for the perceived stress assessment was adapted from the standardized, validated Bruce protocol for clinical exercise stress testing.

**Table 2:** Baseline blood plasma and salivary variables.

Variable	Mean ± SD (normal range)
Plasma glycosylated hemoglobin A1C (%)	5.44 ± 0.38 (5-6)
Plasma glucose (mg/dL)	89.6 ± 14.47 (74-113)
Plasma triglyceride (mg/dL)	98 ± 63.11 (<45-180)
Plasma high-density lipoprotein (mg/dL)	30 ± 5.66 (20-33)
Plasma low-density lipoprotein (mg/dL)	73.4 ± 44.83 (73-121)
Plasma cholesterol (mg/dL)	137.6 ± 28.47 (114-183)
Salivary cortisol (𝛍g/dL)	0.19 ± 0.07 (0.10-0.26)
Salivary testosterone (𝛍g/dL)	121.4 ± 32.06 (75.14-155.56)

This case series is designed to demonstrate the adaptations of cortisol secretion to the 72-h work shift, with respect to the morning (08:00) and evening (20:00) cortisol measurements. Because of the possibility that the combination of being overweight or obese, sleep deprivation, exposure to traumatic events, and previous work schedule would disrupt the normal circadian rhythm of cortisol secretion, it was necessary to measure both the morning and evening cortisol. Although cortisol is typically reported at its lowest around midnight timeframe, we did not want to disrupt the already limited sleep of the working firefighter subjects. 20:00 was selected as the closest proximation for the estimated dip/low end of the cortisol level.

Salivary cortisol and testosterone, blood glucose and triglyceride concentrations, and blood pressures were sampled at 08:00 and 20:00 on days 1, 2, and 3 and 08:00 of day 4 during the same 72-hour work shift for all subjects. These time points were selected based on the intrinsic HPA axis circadian rhythm and diurnal relationship associated with cortisol, specifically with respect to the CAR. Previous studies have shown a significant positive correlation between plasma triglyceride and ectopic adiposity [12]. Because of the high prevalence of obesity in firefighters, we hypothesized that, like cortisol, plasma triglyceride would exhibit a diurnal variation, and there might be a correlation between the cortisol and triglyceride circadian rhythms. The 08:00 cortisol measurements were used as a surrogate biomarker for the CAR, and the 20:00 cortisol measurements were used to characterize the circadian rhythm. Sleep was self-reported and measured by SleepWatch software (Bodymatter Inc.) on an Apple Watch. Sleep disruption was quantified by the number of hours and the number of wake-ups which were negatively correlated (Pearson's coefficient r= -0.94, P< 0001). Periods of active emergency response were documented by the fire department’s automated station log. Table 3 shows the sleep interruption data for the study group. Perceived stress was measured twice daily at 08:00 and 20:00 by requiring each subject to rate their stress from 0 to 10 by matching their perception with a green-to-red color-coded list using the following scale: 0 no stress, 0.5 just noticeably stressed, 1 very light stress, 2 light stress, 3 moderate stress, 4 somewhat heavy stress, 5 heavy stress, 6 intermediate between heavy stress and very stressed, 7 very stressed, 8 and 9 intermediates between very stressed and very very stressed, 10 very very stressed (maximum stress).

**Table 3:** Sleep interruption data.

		Sleep (hours)	Call Volume (number)	Wakeups (number)
Subject 1	Day 1	0.75	15	7
	Day 2	5.25	15	2
	Day 3	4.33	12	3
Subject 2	Day 1	0.75	15	7
	Day 2	5.25	15	2
	Day 3	4.33	12	3
Subject 3	Day 1	0.75	15	7
	Day 2	5.25	15	2
	Day 3	4.33	12	3
Subject 4	Day 1	2.17	18	5
	Day 2	4	13	3
	Day 3	3.5	9	2
Subject 5	Day 1	2.17	18	5
	Day 2	4	13	3
	Day 3	3.5	9	2

Statistical analysis

Salivary cortisol, testosterone and blood glucose and triglyceride concentrations, systolic blood pressure, and perceived stress were quantified. The significance of differences was assessed independently for the seven data collection times over the 72-hour shift as a group (n=5). The nonparametric Friedman test was used to determine the significance of within-group differences using the Wilcoxon-Signed Ranked pairwise comparisons to identify the significance of the specific measurement time points. The goodness of fit for linear regression was used to determine individual trends or correlations between time points. Statistical significance was set at P=0.05 (two-tailed) for all analyses. The linear regression statistic R^2^ was used to identify significant AM and PM trends; P=0.4 was considered significant. In regression analysis, the commonly used criterion for statistical significance is a P-value of less than 0.05. However, it has been argued that this threshold may be too lenient, particularly in studies with large sample sizes, leading to false positive results. A proposed alternative to the p-value threshold is to use a cutoff for regression coefficients, which represent the magnitude and direction of the association between the independent and dependent variables. A commonly used cutoff is a regression coefficient of 0.4 or higher, which has been suggested to represent a moderate to large effect size in a heterogeneous cohort of human subjects [13]. A simulation study demonstrates that a regression coefficient of 0.4 has a high positive predictive value for detecting true associations in a large sample size. This suggests that using a higher threshold for regression coefficients may increase the accuracy of detecting true associations and decrease the likelihood of false positive results. Friedman analysis of variance (ANOVA) and Wilcoxon-Signed Ranked Test pairwise comparisons were performed using SPSS version 24 (IBM, Armonk NY, USA). All descriptive statistics and linear regression correlations were performed using Microsoft Excel.

Case 1: Absence of adaptive downward trend in morning cortisol and adaptive downward trend in evening cortisol

Case 1 is a 30-year-old obese firefighter with hypertension characterized by a high resting heart rate, with high systolic and diastolic blood pressure associated with normal pulse pressure. High resting heart rate x systolic blood pressure product indicates high myocardial oxygen demand. The subject’s 08:00 morning salivary cortisol values were generally higher than his 20:00 evening cortisol, except for day 2 when they were approximately equal. Only a small percentage (18%) of the downward trend in morning cortisol is explained by his diurnal variation (R^2^=0.18) whereas the diurnal variation explains a high percentage (81%) of his evening cortisol (R^2^=0.81). There was a large decrement in both morning and evening cortisol between days 1 and 2 of the three-day work shift. This subject's average morning cortisol was lower than that of the other four subjects (0.142 ug/dL) and his HbA1c was higher (6.0%), in the prediabetic range.

**Figure 1:**
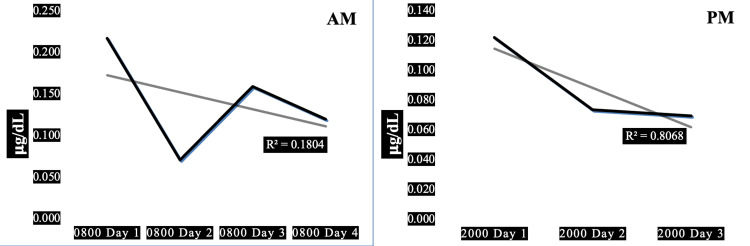
Diurnal morning and evening variations of salivary cortisol in a 30-year-old obese male firefighter. Panel AM: Diurnal variation in morning salivary cortisol showing a trendline of insignificant negative slope (R^2^=0.18). Panel PM: Diurnal variation in evening salivary cortisol showing a trendline of negative slope (R^2^=0.81). Subject characteristics: height 74 in, weight 289 lbs, body mass index 37 kg/sq m, waist-hip ratio 1.08, pulse 81/min, blood pressure 132/90 mmHg, pulse pressure 42 mmHg, years in fire service 12 y, hours worked prior to shift 48 h, hours worked in past 30 days 600 h.

Figure 1 shows the AM and PM changes in salivary cortisol for case 1, one of three firefighters found to exhibit adaptive downward trends in evening cortisol; however, this subject with the absence of an adaptive downward trend in his morning cortisol. Unlike case 2, with adaptive trends in both his morning and his evening cortisol, this subject exhibits an absence of an adaptive downward trend in his morning cortisol. And unlike case 5, with a maladaptive upward trend in morning cortisol, this subject also shows an adaptive downward trend in his morning cortisol.

Case 2: Adaptive downward trends in both morning and evening cortisol

Case 2 is a 40-year-old obese firefighter with hypertension characterized by a normal resting heart rate combined with high systolic and diastolic blood pressure, associated with normal pulse pressure. Normal resting heart rate x systolic blood pressure product indicates normal myocardial oxygen demand. His 08:00 morning salivary cortisol values were higher than his 20:00 evening cortisol, with an approximate 50% decrement between days 1 and 2. A moderately significant percentage (40%) of the downward trend in morning cortisol could be explained by the diurnal variation (R^2^=0.40) whereas an extremely large, highly significant percentage (98%), virtually all this man’s evening cortisol, is explained by his diurnal variation (R^2^=0.98). The decremental trend in morning cortisol is about 50% but is only about 33% for evening cortisol. This subject exhibits an average morning cortisol close to the median, thereby ranking third of the five subjects (0.178 ug/dL) and the second highest HbA1c (5.6%), in the high range of normal.

**Figure 2:**
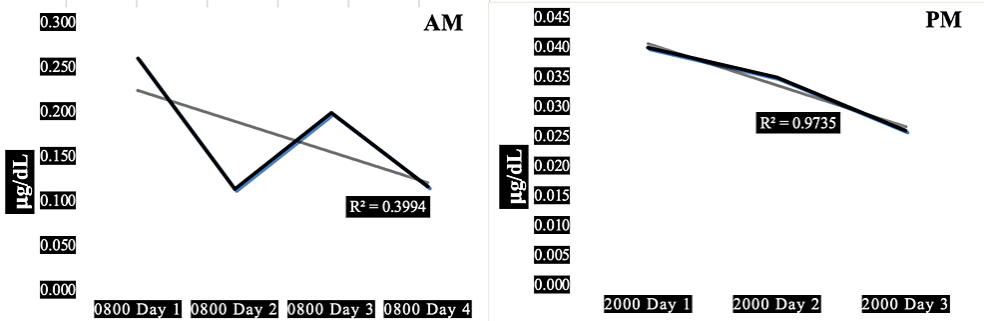
Diurnal morning and evening variations of salivary cortisol in a 40-year-old obese male firefighter. Panel AM: Diurnal variation in morning salivary cortisol showing a trendline of negative slope (R^2^=0.40). Panel PM: Diurnal variation in evening salivary cortisol showing a trendline of negative slope (R^2^=0.98). Subject characteristics: height 73 in, weight 272 lbs, body mass index 36 kg/sq m, waist-hip ratio 0.83, pulse 67/min, blood pressure 130/90 mmHg, pulse pressure 40 mmHg, years in fire service 18 y, hours worked prior to shift 168 h, hours worked in past 30 days 168 h.

Figure 2 shows the AM and PM changes in salivary cortisol for the second of three firefighters exhibiting an adaptive downward trend in evening cortisol; Unlike case 1, with the absence of an adaptive trend in morning cortisol, this subject exhibiting an adaptive downward trend in his morning cortisol. And unlike case 5, with a maladaptive upward trend in morning cortisol, this subject also showing an adaptive downward trend in his morning cortisol.

Case 3: Absence of adaptive downward trends in both morning and evening cortisol

Case 3 is a 31-year-old obese firefighter with hypertension characterized by a moderately elevated resting heart rate combined with high systolic and normal diastolic blood pressure associated with high pulse pressure. High resting heart rate x systolic blood pressure product indicates high myocardial oxygen demand. His 08:00 morning salivary cortisol values were higher than his 20:00 evening cortisol, with inconsistent saw-toothed increments and decrements between days 1 and 3. A very small amount, only 7-9% of the trends in salivary cortisol can be explained by diurnal variation (R^2^=0.07-0.09). This subject exhibits the highest average morning cortisol compared to the other four subjects (0.280 ug/dL) and his HbA1c ranks third, closest to the median value for the cohort (5.4%, normal).

**Figure 3:**
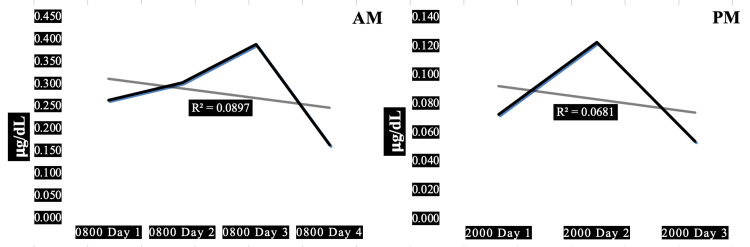
Diurnal morning and evening variations of salivary cortisol in a 31-year-old obese male firefighter. Panel AM: Diurnal variation in morning salivary cortisol showing a trendline of slight, insignificant negative slope (R^2^=0.09). Panel PM: Diurnal variation in evening salivary cortisol showing a trendline of insignificantly negative slope (R^2^=0.07). Subject characteristics: height 72 in, weight 260 lbs, body mass index 35 kg/sq m, waist-hip ratio 0.81, pulse 78/min, blood pressure 134/80 mmHg, pulse pressure 54 mmHg, years in fire service 3 y, hours worked prior to shift 0 h, hours worked in past 30 days 336 h.

Figure 3 presents the AM and PM changes in salivary cortisol for one of five firefighters demonstrating the absence of significant adaptive downward trends.

Case 4: Absence of adaptive downward trend in morning cortisol with maladaptive upward trend in evening cortisol

Case 4 is a 31-year-old morbidly obese normotensive firefighter with elevated resting heart rate, normal systolic, and elevated diastolic blood pressure, associated with low pulse pressure. High resting heart rate x systolic blood pressure product indicates high myocardial oxygen demand. His 08:00 morning salivary cortisol values are generally within the same range as his 20:00 evening cortisol values, with inconsistent saw-toothed increments and decrements in morning cortisol, exhibiting a steep positive trend toward increased evening cortisol between days 1 and 3 of the work shift. A very small amount, only 2%, of the trend in morning salivary cortisol can be explained by this man’s diurnal variation (R^2^=0.02) but a very large percentage (93%) of his evening cortisol can be explained by the diurnal changes (R^2^=0.93). This subject has the second lowest average morning cortisol compared to the other four subjects (0.155 ug/dL) and also the second lowest HbA1c (5.2%, normal).

**Figure 4:**
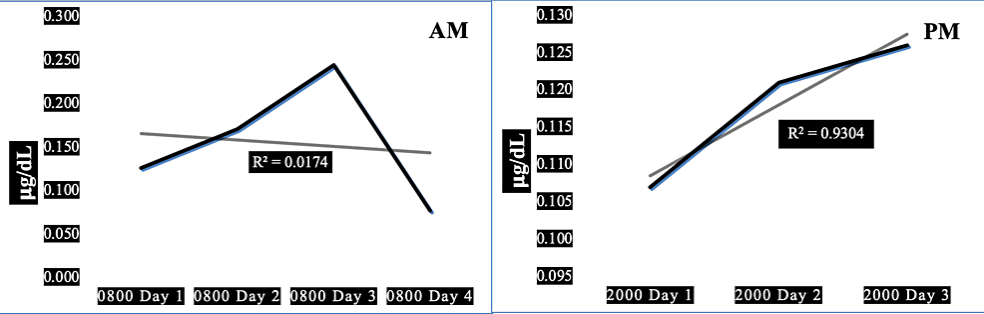
Diurnal morning and evening variations of salivary cortisol in a 23-year-old morbidly obese male firefighter. Panel AM: Diurnal variation in morning salivary cortisol showing a trendline of slight, insignificant negative slope (R^2^=0.02). Panel PM: Diurnal variation in evening salivary cortisol showing a trendline of highly significantly positive (R^2^=0.93). Subject characteristics: height 70 in, weight 341 lbs, body mass index 45 kg/sq m, waist-hip ratio 0.89, pulse 89/min, blood pressure 118/88 mmHg, pulse pressure 30 mmHg, years in fire service 0.5 y, hours worked prior to shift 0 h, hours worked in past days 144 h.

Figure 4 shows the changes in salivary cortisol for one of five firefighters demonstrating the absence of significant adaptive downward trends in AM cortisol with maladaptive upward trend in PM cortisol.

Case 5: Maladaptive upward trend in morning cortisol with adaptive downward trend in evening cortisol

Case 5 is a 23-year-old normal-weight normotensive firefighter with elevated resting heart rate, high systolic and very low diastolic blood pressure associated with very high pulse pressure. High resting heart rate x systolic blood pressure product indicates high myocardial oxygen demand. His 08:00 morning salivary cortisol values were generally markedly higher than his 20:00 evening cortisol values. Large proportions, 91% and 78%, respectively, of the trends in morning and evening salivary cortisol can be explained by his diurnal variation (R^2^=0.91 and 0.78). This subject exhibits the second-highest average morning cortisol compared to the other four subjects (0.254 ug/dL) and the lowest HbA1c (5.0%, normal).

**Figure 5:**
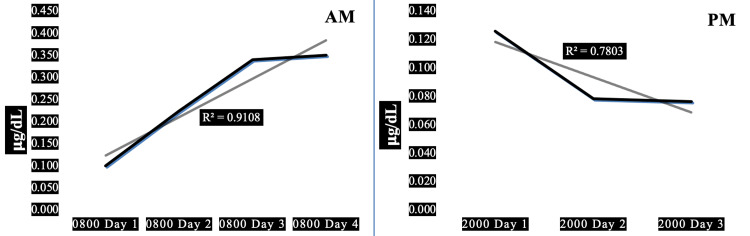
Diurnal morning and evening variations of salivary cortisol in a 23-year-old normal-weight male firefighter. Panel AM: Diurnal variation in morning salivary cortisol showing a trendline of significant positive slope (R^2^=0.91). Panel PM: Diurnal variation in evening salivary cortisol showing a trendline of significant negative slope (R^2^=0.78). Subject characteristics: height 70 in, weight 163 lbs, body mass index 23 kg/sq m, waist-hip ratio 0.88, pulse 80/min, blood pressure 132/58 mmHg, pulse pressure 74 mmHg, years in fire service 1 y, hours worked prior to shift 144 h, hours worked in past days 408 h.

Figure 5 depicts the AM and PM changes in salivary cortisol for one of five firefighters demonstrating a significant maladaptive upward trend in morning cortisol with a significant adaptive downward trend in evening cortisol.

Diurnal variation in salivary cortisol for the firefighter case series

**Figure 6:**
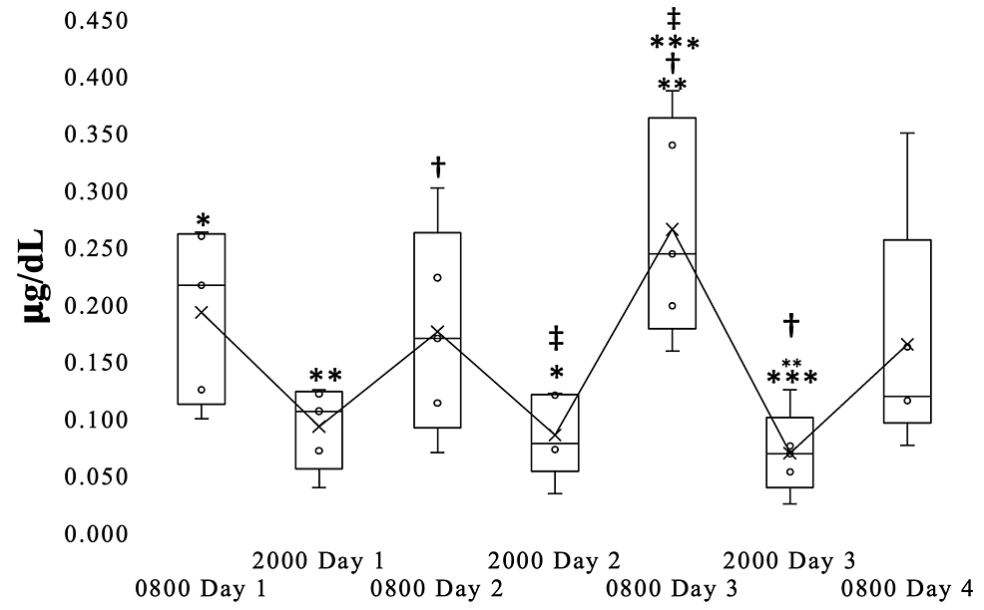
Diurnal variation in salivary cortisol for the firefighter case series. Box plot showing the diurnal variation in cortisol concentrations found in the saliva of the study subjects measured twice daily at 08:00 and 20:00 during the same 72-hour work shift. The lengths of each box indicate the 95% confidence intervals. Within each box, horizontal lines indicate the medians, x’s connected by lines indicate the means. Bars extending from each box indicate the maximum and minimum values. Markers indicate pairwise comparisons significantly different at P< 0.05.

Figure 6 shows the expected, physiological diurnal variation in salivary cortisol concentrations for the 72-hour study period, higher in the AM and lower in the PM (Friedman’s rank-sum test P=0.01). The mean morning cortisol concentration at 08:00 on day 3 was significantly higher than the cortisol concentrations at 08:00 on days 1, 2, and 4.

Relationship between salivary cortisol and perceived stress

**Figure 7:**
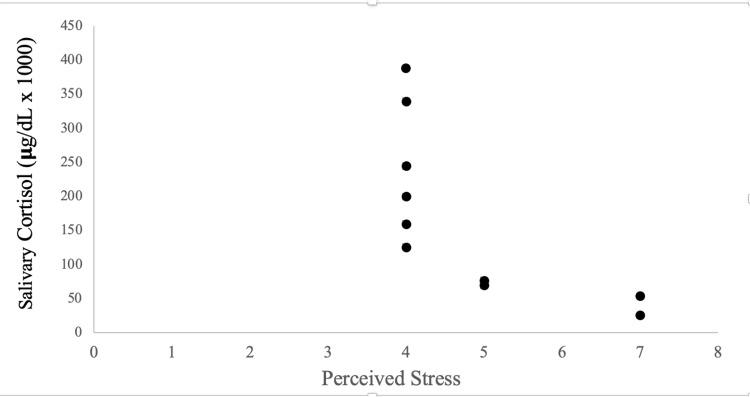
Relationship between salivary cortisol and perceived stress on day 3 of the three-day work shift. Dots indicate perceived stress scores and corresponding salivary cortisol concentrations for each of the 10 measurements performed twice daily at 08:00 and 20:00 on day 3 of the same 72-hour work shift. Pearson product-moment correlation coefficient r = - 0.70, P=0.02. Perceived stress was measured by requiring each subject to rate their stress from 0 to 10 by matching their perception with a green-to-red color-coded list using the following scale: 0 no stress, 0.5 just noticeably stressed, 1 very light stress, 2 light stress, 3 moderate stress, 4 somewhat heavy stress, 5 heavy stress, 6 intermediate between heavy stress and very stressed, 7 very stressed, 8 and 9 intermediates between very stressed and very very stressed, 10 very very stressed (maximum stress).

Figure 7 depicts the salivary cortisol measurements and perceived stress scores for day 3 of the 72-hour study period. A significant correlation was found, r=0.70, P=0.02.

The correlation between perceived stress and salivary cortisol was not significant for days 1 and 2 of the three-day work shift.

Circadian variations in salivary testosterone, plasma glucose, plasma triglyceride, and systolic blood pressure

Circadian variation of salivary testosterone exhibits a trend toward significance (P=0.1, AM>PM). The blood glucose (P=0.02) and triglyceride (P=0.002) assays show significant circadian variations (AM<PM); the normal diurnal variation in systolic blood pressure (AM>PM) was not significant (P=0.2). Elevations in systolic blood pressure were found at each of the time points studied during the 72-hour work shift.

## Discussion

The present case series is designed to demonstrate the patterns of salivary cortisol and testosterone, blood triglyceride and glucose, and systolic blood pressure responses resulting from normal work-related stress in firefighters. The work-related stress was provoked by a combination of the stressful situations and workload and sleep deprivation in a small cohort of four obese, compared to one normal-weight, male firefighters during the same 72-hour work shift. For the purposes of this case series, we defined healthy physiological adaptation as a regression statistic R^2^≥ 0.4, indicating the steepness of a negative slope over the 72-hour, three-day work shift, measured at 8:00 AM or 8:00 PM. The negative slope shows the speed at which the stress-related elevations in salivary cortisol, a positive correlate of the free cortisol concentration in plasma, have declined toward their baseline values for each subject. The main finding of this case series is that the normal-weight subject’s 72-h AM CAR adaptation response was found to be significantly incremental (R^2^=0.91), markedly different than the responses of the four overweight or obese participants who exhibited either flat response (R^2^< 0.4, 3/5 or 60% of the subjects) or decremental response (R^2^=0.40, 1/5 or 20% of the subjects). The predominant 72-hour PM cortisol adaptation was found to be decremental (R^2^=0.78-0.97), in three of the five participants (60%), including the normal-weight participant (R^2^=0.78).

For the cohort, the day 3 CAR cortisol concentration 30 min after awakening was significantly higher than the days 1, 2, and 4 CAR cortisol levels. Augmentation of the CAR on day 3 most likely resulted from overactivation of the HPA axis, known to be the body’s “stress meter.” The stress response begins with the hypothalamic secretion of corticotrophin-releasing hormone (CRH) which, in turn, activates the sympathetic branch of the autonomic nervous system. Salivary cortisol and testosterone were measured simultaneously to determine the relationship between an elevated CAR on day 3 and diurnal variation in testosterone. The physiological circadian rhythm of testosterone secretion is, like the diurnal variation of cortisol, higher in the morning and lower in the evening [14]. It was beyond the scope of this study to investigate the mechanism underlying the association between physiological diurnal salivary cortisol variation and the absence of normal diurnal salivary testosterone variation. However, sympathetic inhibition of the hypothalamic-pituitary-gonadal (HPG) axis which regulates testosterone secretion in males is part of the normal physiological stress response [15]. Sympathetic overactivation, evidenced by elevated systolic blood pressures for all time points studied, is therefore the most likely explanation for the suppression of normal diurnal variation in testosterone. It is noteworthy that, unlike the blunted diurnal testosterone variation, there was no blunting of the expected diurnal blood glucose, lower in the morning and higher in the evening, as well as the diurnal blood triglyceride variation. The blood glucose and triglyceride levels were, largely within the normal range, as were the HbA1c levels. HbA1c was in the prediabetic range (6.0%) for only one subject, the others ranged from 5.0 to 5.6%. Sympathetic overactivation is expected to increase blood glucose [16], an effect not observed in these firefighters during the 72-hour work shift. Sympathetic overactivation is also expected to increase blood lipids by complex lipogenic and lipolytic mechanisms, favoring the mobilization of fats from white adipose tissue [17]. Such adipokinesis is mediated by the catecholamines released with sympathetic stimulation of white adipose tissue and liver [18]. The median blood triglyceride levels found in this case series were, generally, within the normal range for males of the age of the subjects, thereby suggesting an (unexplained) absence of a sympathetic effect on blood glucose and triglycerides. These findings are supported by individualized discussions of the cases.

Case 1 presents a pattern of salivary cortisol likely dominated by a combination of sympathetic overactivity and metabolic syndrome. This is evidenced by the high resting heart rate, blood pressure, body mass index, and waist-hip ratio, the latter indicative of abdominal obesity. The main finding related to case 1 is that the downward trend in the morning cortisol does not, but the downward trend in his evening cortisol does, explain a large percentage of the salivary cortisol throughout the 72-h work shift. A large decrement in both morning and evening cortisol between days 1 and 2 of the three-day work shift in this firefighter may be explained by significantly more hours of sleep on day 2 than on day 1 of the 72-h work shift (0.75 h vs. 5.25 h, Table 1). Similarly, the downward trends in both morning and evening cortisol may reflect relatively more sleep on day 2 and day 3 than on day 1 (0.75 h < 5.25 h and 4.33 h on days 2 and 3, respectively).

Case 2 demonstrates a pattern of salivary cortisol not likely dominated by sympathetic overactivity, but more likely by other, perhaps metabolic, or genetic, factors. This is evidenced by the normal resting high heart rate, despite high blood pressure. This subject’s large body mass index with a relatively normal waist-hip ratio suggests the absence of metabolic syndrome as an explanation for his diurnal cortisol variations. The main finding related to case 2 is that the downward trends in both the man’s morning and evening cortisol explain a very large percentage of his salivary cortisol throughout the 72-h work shift. The large 50% decremental trend in morning cortisol in this firefighter may be explained by significantly more hours of sleep on day 2 than on day 1 of the 72-h work shift (0.75 h vs. 5.25 h, Table 1). However, the added sleep appears to be less effective at decreasing his evening cortisol. Thus, there may be other factors at play in blunting the evening cortisol drop-off throughout the three-day work shift.

Case 3 exhibits a pattern of salivary cortisol likely resulting from a combination of sympathetic overactivity and low arterial compliance. This is evidenced by the elevated heart rate, systolic hypertension in the absence of diastolic hypertension, which serves to widen his pulse pressure. Such cardiovascular dynamics are consistent with increased aortic-arterial pulse wave velocity associated with aortic and another large arterial stiffening which is likely to limit diastolic runoff from large to small arteries blood flow to peripheral capillary beds, as well as venous return for maintaining cardiac output. The large body mass index with a relatively normal waist-hip ratio suggests the absence of metabolic syndrome as an explanation for his diurnal cortisol variations. The main finding related to case 3 is the absence of downward trends in both the morning and the evening cortisol suggestive of a failure of this firefighter’s cortisol responses to adapt to the three-day work shift.

Case 4 shows a pattern of salivary cortisol likely resulting from a combination of sympathetic overactivity with normal arterial compliance. This is evidenced by elevated heart rate with normal systolic and high diastolic blood pressure which serves to narrow the pulse pressure. Such cardiovascular dynamics are consistent with decreased aortic-arterial pulse wave velocity likely associated with the high heart rate, fast diastolic blood flow runoff to small arteries and capillaries, and/or high systemic arterial (total peripheral) resistance for maintaining venous return and cardiac output. The very large body mass index with a normal waist-hip ratio suggests the absence of metabolic syndrome as an explanation for his diurnal cortisol variations. The main finding related to case 4 is the absence of downward trend in morning cortisol suggestive of a failure of this firefighter’s morning cortisol responses to adapt to the three-day work shift, but with a marked maladaptive pattern of increasing evening cortisol secretion throughout the work shift. The absence of an adaptive downward trend in AM cortisol is likely to result in elevated AM cortisol levels. Elevated AM cortisol has been associated with immune suppression which limits the capacity for recovery from illnesses in nurses and other shift workers [19]. Elevated AM cortisol can lead to insulin resistance, increasing the risk of developing type 2 diabetes and metabolic syndrome [20] and increased risk of hypertension, atherosclerosis, and cardiovascular events [21]. Elevated AM cortisol can also contribute to the development of anxiety, depression, and PTSD, as well as exacerbating pre-existing mental health conditions [22].

Case 5 presents a pattern of salivary cortisol likely resulting from a combination of sympathetic overactivity with high arterial compliance. This is evidenced by elevated heart rate with high systolic and very low diastolic blood pressure which serves to markedly increase the pulse pressure. Such cardiovascular dynamics are consistent with increased aortic-arterial pulse wave velocity likely associated with high arterial compliance and fast diastolic blood flow runoff to small arteries and capillaries, and/or high systemic arterial (total peripheral) resistance for maintaining venous return and cardiac output. The normal body mass index with a normal-high waist-hip ratio suggests the possibility of metabolic syndrome as an explanation for his diurnal cortisol variations. The main finding related to case 5 is the presence of upward trend in morning cortisol suggestive of maladaptation of this firefighter’s morning cortisol responses, but with a physiological adaptive pattern of decreasing evening cortisol secretion throughout the work shift. This firefighter exhibits a maladaptive upward trend in morning cortisol, indicating a dysregulated stress response. However, the adaptive downward trend in evening cortisol suggests that the subject's HPA axis is able to recover and regulate itself throughout the day. This dysregulation of the stress response system could potentially lead to the previously mentioned negative long-term health outcomes in nurses and other shift workers such as the increased risk of metabolic disorders, cardiovascular disease, and mental health issues. Further studies should address these potential risks in firefighters exposed to repeated sleep deprivation and traumatic events during 72-hour work shifts.

Limitations

This case series is intended to elucidate the patterns of change in cortisol in firefighters over a 72-hour work shift specifically because such changes have not been previously explored. The small sample size of this case series is a major limitation, which makes it difficult to generalize the findings to a larger population. Because of the small N, the findings are statistically underpowered, and interpretations derived therefrom are prone to type 2 statistical errors. Although the small sample size may not be statistically representative of the US firefighter population at large, the five firefighters studied were members of the largest fire department in California and are representative of the standard American firefighter lifestyle (non-smoking, 1-hour physical training/day, social drinking, and overweight or obese). The lack of a control group limits the ability to draw firm conclusions about the effects of sleep interruptions on cortisol levels. The method used to quantify stress was also limited to self-report, which may be subject to bias and variation among individuals. Future studies should consider using a validated questionnaire of stress perception to control for subjectivity. Additionally, the surrogate marker used to measure the CAR is untested and should be validated in future studies. The study design also created conditions for finding roughly the same number of sleep interruptions, which may have affected the ability to detect significant correlations between cortisol levels and sleep quality. Also, the patterns of diurnal cortisol variations observed may also be influenced by factors such as obesity and extensive work shift hours, which were not fully controlled for. While this case series provides important insights into the potential effects of sleep interruptions on cortisol levels in firefighters, the limitations of the study design and sample size suggest that further research is needed to confirm and extend these findings. This case series represents the first, albeit limited, examination of a 72-hour firefighter shift experience, the longest and potentially most detrimental to first responders. During this particular 72-h work shift, the firefighter subjects experienced critical call exposure/response, structure fire with vertical ventilation, drug overdoses, adolescent suicide, as well as sleep disruption which negatively impacts thousands of firefighters and shift workers every year by increasing the incidence of serious and debilitating disease etiologies. The P<0.05 criterion for significance was reached for the main findings of the case series concerning the cortisol diurnal rhythm and related adaptations, suggesting significant physiological phenomena rather than the effects of type 1 statistical error. Our surrogate marker for the CAR is untested and its validation should be the subject of a future case series or study. The case series design created the conditions for finding roughly the same number of interruptions and durations of sleep. Significant statistical correlations require two variables to change proportionality. The salivary cortisol exhibited normal physiological variability, higher in the morning and lower in the evening whereas the variability in sleep duration and interruptions were slight, thereby not fulfilling the main criterion for correlation. There was no control group of firefighters without sleep interruptions for comparison to the case study group with sleep interruptions. The relatively short, 72-hour length of the work shift did not allow us to determine each subject’s baseline diurnal rhythms for the variables studied, before the 72-hour work shift. Future investigations should, therefore, include a control group and a baseline period of the same duration as the work shift under investigation. Cortisol in class 1 and 2 obesity is known to vary and could be an independent factor for explaining the salivary cortisol variations in this case series cohort. The patterns of diurnal cortisol variations described herein might also be related to the extensive work shift hours logged. Nevertheless, no participant had any prior medical history, diagnosis, or prescription that could have affected cortisol levels. This was part of the criteria included in the recruitment flyer and confirmed by each individual participant via signed consent. To our knowledge, this is the first study to investigate cortisol levels during a 72-hour work shift in sleep-deprived firefighters so there is no current published literature to compare or evaluate “expected ranges” or normal vs abnormal cortisol levels for this population over this time period.

Physiological correlates of the cortisol adaptations

This case series reports the diurnal variation in salivary cortisol in firefighters during a 72-hour shift. We expected to find a correlation between cortisol levels and the amount of sleep disruption; however, we found none. Cortisol secretion is reported to be elevated in several cohorts of sleep-deprived shift workers [18,23-28] including firefighters [29,30]. We expected to find a significant positive correlation between salivary cortisol levels and perceived stress like what has been reported in Saudi dental students during periods of academic exams [31], but we found a significant negative correlation, only on day 3 at the end of the 72-hour shift. Perceived stress had no effect on cortisol in a large Danish cohort of public service employees [32]. A negative correlation between cortisol and perceived stress during cold pressor testing (cold stress) has been demonstrated [33]; although, the mechanism remains unclear. Cold pressor testing is known to produce a robust painful stimulus that strongly activates sympathetic nerves mainly systemic arterioles [34]. It is plausible that this negative correlation between perceived stress and salivary cortisol reflects psychological and/or physiological adaptations to repeated exposure to environmental stressors in the three veteran firefighters with 3-12 years in service: two of the five subjects with only six months and one year of firefighting service, respectively. All our subjects exhibited strong vasopressor activity during their 72-hour shift, evidenced by their elevations of blood pressure, which might have increased their cerebral blood flows and mood states, thereby serving to alter their perceptions of stress [35]. Their perceptions of stress were also likely to have been influenced by the extensive prior work shift hours.

One of the more interesting aspects of this case series is the absence of a normal nighttime “dipping” in systolic blood pressure which is a physiological indicator of the risk for hypertension [36]. Sympathetic overactivity is the most plausible explanation. The subjects had no histories of diagnoses or treatments for hypertension. It is, therefore, likely that the elevated systolic blood pressures and absence of nighttime dipping were part of the subjects’ response to the 72-hour shift work. However, we cannot rule out the possibility that they had previously undiagnosed chronic hypertension. Indeed, four of the five subjects were, by body mass index standards, overweight or obese - one of the more significant risk factors for hypertension. The lack of correlation between cortisol levels and blood pressure, as previously mentioned for the relationship between cortisol and sleep disruption, can be explained by the relatively small diurnal variations in systolic blood pressure during the 72-hour shift despite the blood pressure elevation. Whether these findings represent physiological adaptations to years of shift work with sleep disruptions in the three of five veteran firefighters in this case series remains unclear; however, it suggests hypotheses concerning differences between novice and veteran firefighters which may be worthy of further investigation.

This case series demonstrates, for the first time, the diurnal/circadian variations in cortisol, testosterone, triglyceride, glucose, and blood pressure in a group of firefighters during the course of the same 72-hour work shift. The majority of firefighters in the US workforce and four of the five participants in the present case series were hypertensive and/or obese, one with HbA1c in the prediabetes range. Their diurnal/circadian variations suggest the possibility of maladaptations which may affect their health and firefighting performance. Firefighting operations are physically arduous, difficult, demanding, and often highly stressful. Structure fire standard operating procedures may vary with the authority having jurisdiction and are often more complicated with multiple agency response/mutual aid agreements but the demands of the job do not change. Structure turnout gear/personal protective equipment (PPE) is designed to keep radiant heat from hot smoke and gas out; however, the protective layers prevent effective thermoregulation and body cooling by keeping the heat in. This heat retention contributes to heat injuries such as heat cramps, heat exhaustion, and heat stroke [37]. Obesity further increases susceptibility to heat stress and reduces effective thermoregulation due to blunted body cooling mechanisms [38]. Hose lays, ladder work, forcible entry, interior fire attack, vertical ventilation, rescue operations (often involving extensive crawling and victim dragging), salvage and overhaul are all physically taxing even for normal-weight firefighters, however, obese firefighters are over five times more likely to experience incidence of musculoskeletal injury compared to normal weight colleagues and three times more likely to experience follow-up injuries [39]. Additionally, an estimated 20% reduced work capacity occurs in full PPE on air with self-contained breathing apparatus (SCBA) while operating in hazardous environments, along with intense physical exertion, increased heart rate, blood pressure, and myocardial oxygen demand [40]. Preexisting hypertension independently increases the risk of cardiovascular disease and myocardial infarction; however, with the addition of full PPE and associated heat-trapping, arduous fire ground operations, and obesity the likelihood of a cardiac event resulting in a line of duty death becomes a more dangerous possibility [41]. There is a 50-70% increased risk of job-related disability resulting in lost work time directly associated with obesity in male firefighters. Lost work time includes sick leave used for acute and chronic injury or illness and associated early resignation/retirement, involuntary termination, and/or death [42].

## Conclusions

This case series was designed to compare the CAR-related response characteristics of a normal-weight firefighter with those of four obese firefighters. The aim was to identify critical differences and factors which may be useful for identifying firefighters at high risk for developing stress-related cardiovascular disease and suicide-related behaviors. The ultimate goal is to motivate the development of an effective intervention to decrease the prevalence of stress-related diseases and suicide in firefighters, targeting the highest-risk individuals. The findings of this case series should be interpreted cautiously pending a study with adequate statistical power. Nevertheless, this small case series provides new insights into how the diurnal variation in salivary cortisol may be affected in firefighters subjected to a 72-hour work shift. We found that three of the five subjects (60%) exhibited day-to-day decremental CAR responses during the 72-h work shift, representing what would be expected to occur as normal physiological adaptation. The day 3 awakening response cortisol concentration 30 min after awakening was elevated significantly out of the normal range. Day 3 awakening response cortisol levels were negatively correlated with perceived stress. Physiological diurnal variations in salivary cortisol, blood glucose, and triglyceride were present but physiological diurnal variations in testosterone and systolic blood pressure were absent. The elevations in systolic blood pressure found at each of the time points studied during the 72-hour work shift suggest stress-induced overactivation of the sympathetic branch of the autonomic nervous system as the primary cause. Such autonomic imbalance may put firefighters at increased risk for developing a wide variety of stress-related diseases such as hypertension, heart failure, as well as neuropsychiatric disorders, and suicidal behaviors. The non-decremental, non-incremental “flat” CAR adaptation observed in two of the five subjects is a cortisol signature that has been previously reported for survivors of suicide. It is hoped that the findings of this case series will motivate and guide future research to evaluate the contributions of the CAR to the development of stress-related diseases and to design effective interventions for the prevention of stress-related diseases and suicide in firefighters.

This case series is the first to describe the variations in diurnal/circadian rhythms of cortisol, testosterone, triglycerides, glucose, and blood pressure in a cohort of firefighters during the course of the same 72-hour work shift. Our main finding is the absence of an adaptive downward trend in AM cortisol characterized by maladaptive flat or upward trends, indicating a dysregulated stress response. The implications of these findings for practice and policy include issues such as the need to eliminate hypertensive, obese firefighters from performing the most arduous physical firefighting tasks. Relegating individuals who fail to meet accepted health and physical fitness standards to administrative duties is likely to help create a culture of wellness in the best interests of both firefighters and of the public they serve. Further studies should address these potential risks in firefighters exposed to repeated sleep deprivation and traumatic events during 72-hour work shifts.
